# Aortic root replacement and valvuloplasty in a woman with severe pectus excavatum

**DOI:** 10.15171/jcvtr.2018.19

**Published:** 2018-05-21

**Authors:** Panagiotis Kalogris, Kosmas Iliadis, Gregory Amanatidis, Alexandros Demis, Anna Smyrli, Konstantinos Petsios

**Affiliations:** ^1^Second Department of Adult Cardiac Surgery, Onassis Cardiac Surgery Center, Athens, Greece; ^2^Director of Thoracic Surgery Clinic, Hygeia Hospital, Athens, Greece; ^3^Nursing Clinical Research Office, Onassis Cardiac Surgery Center, Athens, Greece

**Keywords:** Pectus Excavatum, Aortic Root, Valvuloplasty, Marfan

## Abstract

We describe a case of a 43-year old woman with Marfan’s syndrome, tricuspid valve regurgitation and severe pectus excavatum who required an aortic root replacement and valvuloplasty for an ascending aortic aneurism with aortic valve regurgitation and tricuspid valve regurgitation. There was a severe angulation of the sternum which was close to 1.5 cm to the column vertebrae. Such cases are quite rare and always a challenge for surgeons to achieve adequate exposure of the heart and prevent excessive cardiac compression. We present our access, a safe and efficient exposure of the heart in order to achieve the best outcome.

## Introduction

Severe pectus excavatum repair combined with a cardiac surgery is a quite rare and challenging procedure.^1^ The surgeons face a variety of difficulties concerning the safety of chest exposure and use different approaches. In cases of severe pectus excavatum there is an excessive dislocation of cardiac formation and increased possibility for postoperative chest compression. Modified Ravitch type procedures have been used as a safe and reliable approach in cases of simultaneous repair.^2^ However, many authors still face the dilemma regarding the best time to manage simultaneously these condition.^3,4^

## Case Presentation

**Figure 1:**
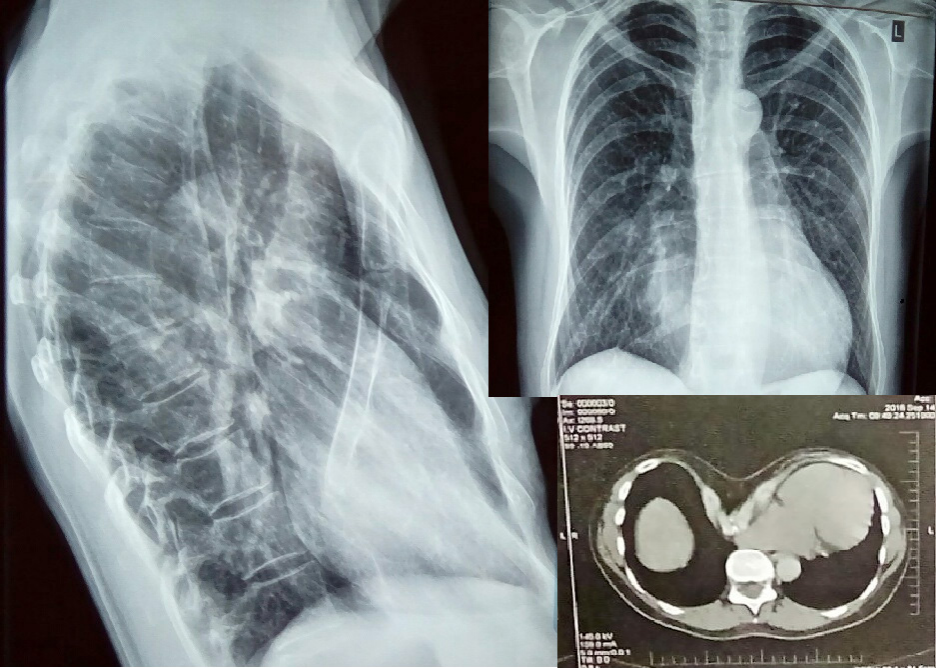
Preoperative Rx and CT images.

A 43-years old woman with Marfan’s syndrome was diagnosed with progressive aortic root enlargement to 5.4 cm, aortic valve regurgitation and tricuspid valve regurgitation. Elective repair was highly recommended. Her clinical examination along with the preoperative RX and CT scan revealed a severe pectus excavatum. Her sternum was angled and was only 1.5 cm close to the column vertebrae at the point of apex. This resulted in a total cardiac dislocation to the left (Figure 1).

Due to the severe sternal deformity, the affinity of the right atrium and the vena cava to the sternum, we enacted the operation with the exposure of the right femoral artery and vein to assure a quick access to cardiopulmonary bypass (CBP) if it was required. After the sternotomy it was very difficult to continue with sternal retractors, even if we tried to use a couple of them in upper and lower sternum. Alternatively, we used an Osler retractor at the left hemisternum to expose ascending aorta and right atrium. A partial CBP was then introduced with the ascending aorta and right atrium cannulation that gradually resulted in a total one with the cannulation of superior and inferior vena cava. Afterwards, a standard valve and aortic root replacement with a re-implantation technique was performed with the use of a 21 cm composite graft and Tricuspid valve annuloplasty with a 32mm physio tricuspid annular ring.

**Figure 2:**
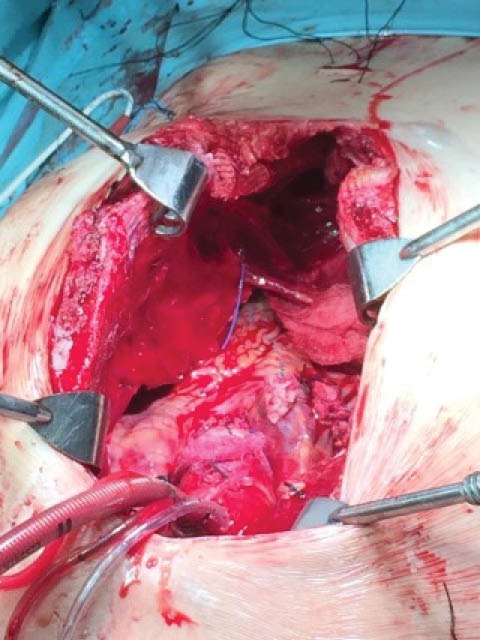
Chest cavity exposure using Osler retractors.

Once the operation was performed and the hemostatic procedures achieved adequate bleeding control, a post-repair transesophageal echocardiography demonstrated normal ventricular function with overall good composite graft function and no tricuspid insufficiency. Then a second Osler retractor was applied to the right hemi-sternum to minimize the compression of the cardiac structure and to achieve a safer disconnection from the CPB (Figure 2). This technique achieved to minimize the danger of excessive chest compression intraoperatively and revealed as an additional aid for Ravitch procedure. After the CPB termination and protamine administration, we carefully checked all surgical sites to ensure that there was no bleeding. Our next step was to perform a Ravitch II procedure. In brief, bilateral pectoralis muscle flaps were raised and the costal cartilages from rid 3 to 8 were resected, taking care to retain the perichondrial sheaths.

**Figure 3:**
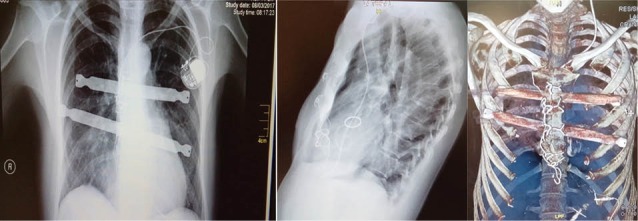
Postoperative Rx and CT images.

Finally, the sternum closure was performed with steel wires and reduction was maintained with two substernal bars. No significant bleeding occurred postoperatively. The patient remained heamodynamic stable during acute ICU care and was extubated 10 hours later. Sub-pectoral drains, as also sub-sternal and hemi-thorax drains were removed on postoperative day three. Her postoperative course was complicated by a trifascicular block that required the placement of a permanent pacemaker on day 9, with a total hospitalization of 11 days. A follow up CT scan demonstrated satisfactory reduction in the pectus deformity with a stable aortic and tricuspid repair. One year later she is in excellent condition and postoperative tests are normal (Figure 3).

## Discussion

The concomitant repair of pectus combined with any type of cardiac surgery in patients with Marfan’s syndrome is challenging.^1^ Moreover, there is a great controversy regarding repair of pectus excavatum in such patients and the choice of adequate chest opening technique.^2,3^ Repair of pectus excavatum after a previous cardiac surgery can be extremely challenging especially due to the postsurgical adhesions between the sternum and heart.^5^ Ravitch type procedures have occasionally been used and authors state that this technique is a safe and reliable approach in cases of simultaneous repair.^1,2^

In the past more invasive procedures have also been described, such as the sternal turnover, eversion in a trapdoor fashion, turnover with internal thoracic-vessel presentation or even a modified Nuss procedure.^1,3,5^ These techniques offer excellent operative exposure but they are associated with a high percentage of wound infection or sternal necrosis, despite the preservation of the internal thoracic blood supply.^1^

In our case the use of Osler retractors was an additional aid for the Ravitch connection of the sternum. The stepped wised Ravitch procedure on the left at first and then on the right was performed in order to avoid any loss of cardiac output after the removal of the retractors due to cardiac compression from the tilt chest wall. Yeung et al described in a similar case a sudden loss of cardiac output after removal of the retractors.^2^

Ravitch correction was performed with precautions to minimize any cardiac complication after the sternal closure. Additionally, a number of previous articles related extended operating time with bleeding, infections and other complications.^3,6^ Therefore, we followed a careful step by step approach in order to ensure that there will be no need for chest reopening postoperatively in order to manage a cardiac or surgical complication.^7^ Indications for simultaneous pectus correction and cardiac surgery include the elimination of a second staged procedure and avoidance of early post-operative cardiac compartment syndrome following the compression from the sternal deformity.^8^ Therefore, we strongly recommend the close cooperation of thoracic and cardiac surgeons for the successful management of such cases.

## Ethical approval

Patient has signed informed consent allowing her information to be used in this case report.

## Competing interests

All authors declare no competing financial interests exist.
